# An Interesting Relic

**Published:** 1867-06

**Authors:** 


*An Interesting Relic.*—Dr. Jas. F. Thompson of Fredericksburg, Va. has presented to the museum of the Baltimore College of Dental Surgery, an entire set of artificial teeth, made during the Colonial times of Virginia.

The teeth are carved from ivory, mounted on gold plates, and connected together by means of springs. That they have been long worn is evident from the number of times they have been rudely repaired, and they are especially interesting as showing the action of deleterious agents upon the ivory, a number of carious cavities existing in the different blocks, together with accumulations of salivary calculus.

